# Angiotensin II Inhibits Insulin-Stimulated GLUT4 Translocation and Akt Activation through Tyrosine Nitration-Dependent Mechanisms

**DOI:** 10.1371/journal.pone.0010070

**Published:** 2010-04-07

**Authors:** Alfredo Csibi, David Communi, Nathalie Müller, Serge P. Bottari

**Affiliations:** 1 Laboratoire de Bioénergétique Fondamentale et Appliquée, INSERM U884, Grenoble Universités, Grenoble, France; 2 IRIBHM, Université Libre de Bruxelles, Brussels, Belgium; 3 CHU de Grenoble, Grenoble, France; McMaster University, Canada

## Abstract

Angiotensin II (Ang II) plays a major role in the pathogenesis of insulin resistance and diabetes by inhibiting insulin's metabolic and potentiating its trophic effects. Whereas the precise mechanisms involved remain ill-defined, they appear to be associated with and dependent upon increased oxidative stress. We found Ang II to block insulin-dependent GLUT4 translocation in L6 myotubes in an NO- and O_2_^.−^-dependent fashion suggesting the involvement of peroxynitrite. This hypothesis was confirmed by the ability of Ang II to induce tyrosine nitration of the MAP kinases ERK1/2 and of protein kinase B/Akt (Akt). Tyrosine nitration of ERK1/2 was required for their phosphorylation on Thr and Tyr and their subsequent activation, whereas it completely inhibited Akt phosphorylation on Ser^473^ and Thr^308^ as well as its activity. The inhibitory effect of nitration on Akt activity was confirmed by the ability of SIN-1 to completely block GSK3α phosphorylation in vitro. Inhibition of nitric oxide synthase and NAD(P)Hoxidase and scavenging of free radicals with myricetin restored insulin-stimulated Akt phosphorylation and GLUT4 translocation in the presence of Ang II. Similar restoration was obtained by inhibiting the ERK activating kinase MEK, indicating that these kinases regulate Akt activation. We found a conserved nitration site of ERK1/2 to be located in their kinase domain on Tyr^156/139^, close to their active site Asp^166/149^, in agreement with a permissive function of nitration for their activation. Taken together, our data show that Ang II inhibits insulin-mediated GLUT4 translocation in this skeletal muscle model through at least two pathways: first through the transient activation of ERK1/2 which inhibit IRS-1/2 and second through a direct inhibitory nitration of Akt. These observations indicate that not only oxidative but also nitrative stress play a key role in the pathogenesis of insulin resistance. They underline the role of protein nitration as a major mechanism in the regulation of Ang II and insulin signaling pathways and more particularly as a key regulator of protein kinase activity.

## Introduction

Increasing evidence from in vitro studies and animal models using ACE inhibitors (ACEI), Ang II AT_1_ receptor antagonists (ARB) and more recently renin inhibitors (RI), indicates that Ang II is involved in insulin resistance [1].

Most important, several clinical trials with ACEI and ARBs have shown that blockade of the renin-angiotensin system not only slows down the progression of cardiovascular morbidity and mortality in type 2 diabetic patients [2] but also reduces the risk of developing diabetes among hypertensive patients (reviewed in [3], [4]). Even more interesting are the observations that both drug families have been shown to increase insulin sensitivity [3], [4], [5], [6], [7], indicating that Ang II directly interferes with insulin-dependent metabolic pathways and therefore probably is involved in the etiology of diabetes.

Despite the clinical implications of these observations, most investigators have focused their efforts on studying the interference of Ang II with insulin signaling pathways in the vascular wall rather than on major metabolic target tissues like the liver, adipose tissue and skeletal muscle [1], [6]. Several reports on the insulin-desensitizing effects of Ang II in skeletal muscle indicate that Ang II reduces insulin-mediated glucose uptake [8], [9], [10], [11], [12] and GLUT4 translocation [9], [11], [13]. On the other hand, Ang II has been shown to stimulate the production of reactive oxygen species (ROS) in endothelial and vascular smooth muscle cells as well as in various tissues including skeletal muscle in various animal models [8], [11], [14] and this pathway has been suggested to play a major role in its insulin desensitizing effects [1], [8], [11], [14], [15].

Although superoxide ions (O_2_^−.^) and hydrogen peroxide (H_2_O_2_) have been reported to affect numerous signaling pathways, the precise molecular mechanisms through which they alter specific enzymatic activities have often not been elucidated. With regard to this question, we have shown previously that ROS-dependent activation of ERK1/2 by Ang II [16] is in fact due to the production of the RNS peroxynitrite (ONOO^−^), which leads to their nitration on tyrosine residues [17]. This highly reactive species results from the reaction of O_2_^−.^ with nitric oxide (NO) and is generated in many pathological conditions which involve oxidative stress, such as diabetes [18], [19], [20]. Peroxynitrite can act both as a strong oxidant, e.g. on lipids which it can peroxidize, and as a nitrating and S-nitrosating agent on proteins, DNA, lipids and fatty acids [20], [21]. It has also been shown to affect the functional properties of several proteins including kinases [20] among which a key enzyme of insulin signaling, Akt [22]. The molecular mechanism through which ONOO^−^ regulates this kinase has however not been investigated so far although Akt has been reported to be S-nitrosated in diabetic mice and in response to NO donors [23], [24], indicating that NO and RNS play an important role in its regulation. As we showed previously that Ang II induces the nitration rather than the S-nitrosation of other kinases [17], implying the activation of pathways generating more ONOO^−^ than required for S-nitrosation [21], we hypothesized that this might also be the case with Akt, especially as it has been recently reported that stimulation of peroxynitrite catalysis restored Akt phosphorylation and insulin-stimulated glucose uptake in insulin-resistant mice [25].

The purpose of this study was to clarify the molecular mechanisms through which Ang II regulates insulin-dependent Akt activity with regard to glucose uptake in a skeletal muscle model, differentiated L6 cells.

We found that Ang II completely blocks insulin-stimulated GLUT4 translocation and strongly inhibits insulin-mediated Akt phosphorylation on Ser^473^ and Thr^308^. This effect of Ang II was ROS- and RNS-dependent. Ang II induced tyrosine nitration of Akt and this nitration inhibited its phosphorylation as well as that of its target protein GSK3α. We also found Ang II-activated ERK1/2 to inhibit insulin-dependent Akt activity. Finally, we mapped two nitration sites of ERK1/2 and found one them to be Tyr^156/139^, located close to the C-loop and the active site Asp^166/149^, potentially in agreement with a permissive function of nitration for their activation.

Taken together these findings indicate that one of the mechanisms through which Ang II decreases insulin sensitivity in skeletal muscle is the inhibition of Akt through at least two RNS-dependent pathways: first, through the transient activation of the MEK-ERK1/2 pathway which inhibits IRS1/2-dependent signaling and second through direct nitration of Akt.

Our data also identify Akt is a novel nitroprotein and further stress the role of peroxynitrite as a major determinant of insulin resistance and hence as a potential pharmacological target.

## Results

### Ang II impairs insulin-mediated translocation of GLUT4

**Figure 1:**
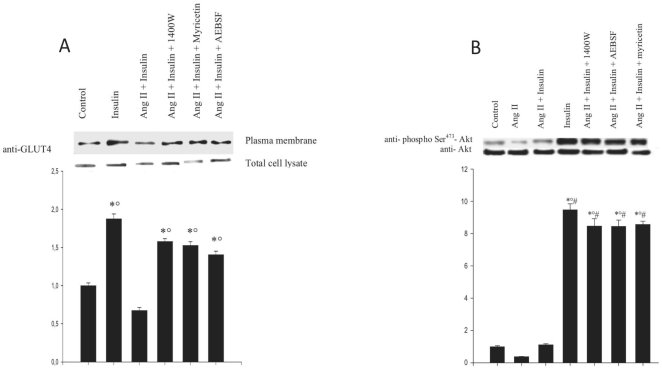
Effect of ROS and RNS modulators on Ang II-regulated insulin-mediated GLUT 4 translocation and Akt phosphorylation. L6 myotubes were incubated with AEBSF (0.5 mM) for 30 min, 1400 W (10 µM) for 60 min and myricetin (100 µM) for 30 min, prior to incubation with Ang II (10 nM) for 30 min and subsequent stimulation with insulin 100 nM for 60 min. ***A***: Blots of the plasma membrane or total cell lysate proteins were probed with anti-GLUT4 antibody. Results are expressed as fold change of untreated control (means ± s.d.) and the blot is representative of three independent experiments (*P<0,05 vs control, °*P*<0,05 vs Ang II + insulin). ***B***: Western blots were probed with anti-phosphoSer^473^-Akt and anti-Akt antibodies. The blot displayed is representative of three independent experiments. Results are expressed as the fold increase of the ratio of phospho-Akt/Akt over controls (means ± s.d.) (*P<0,05 vs control, °*P*<0,05 vs Ang II, ^#^ *P*<0,05 vs Ang II + Insulin).

As shown in Fig.1*A*, insulin (100 nM) stimulated translocation of the glucose transporter GLUT4 to the plasma membrane approximately two-fold after 1 hour. Preincubation of L6 myotubes with Ang II (10 nM) for 30 min prior to stimulation completely abolished insulin-mediated GLUT4 translocation, which decreased below control levels (P<0.05). The strength of this inhibition suggests that blockade of this glucose transporter's translocation is the main mechanism through which Ang II inhibits glucose uptake in skeletal muscle.

In order to test whether this action of Ang II involves a ROS- or RNS-dependent pathway, the effect of compounds interfering with the generation of these reactive species on GLUT4 translocation was investigated. As shown in the same panel, the Ang II-mediated inhibition of insulin-dependent translocation was significantly (P<0.05) alleviated by the NADP(H) inhibitor AEBSF (0.5 mM), the selective iNOS inhibitor 1400 W (10 µM) and the O_2_^−.^ scavenger myricetin (100 µM), indicating that both NO and O_2_^−.^ generation are involved in this signalling cascade. These compounds had no significant effect per se on GLUT4 translocation under control or insulin-stimulated conditions but abolished the effect of Ang II alone (data not shown).

### Ang II impairs insulin-mediated phosphorylation of Akt

As Akt is considered to be the upstream regulator of GLUT4 translocation we tested the effect of Ang II on its activation in response to insulin. This was achieved by determining its level of phosphorylation on Ser^473^ and Thr^308^ residues which is known to be required for its full activation by the upstream kinases PDK1 and mTORC2 and subsequent activation of AS160 which triggers GLUT4 translocation.

**Figure 2:**
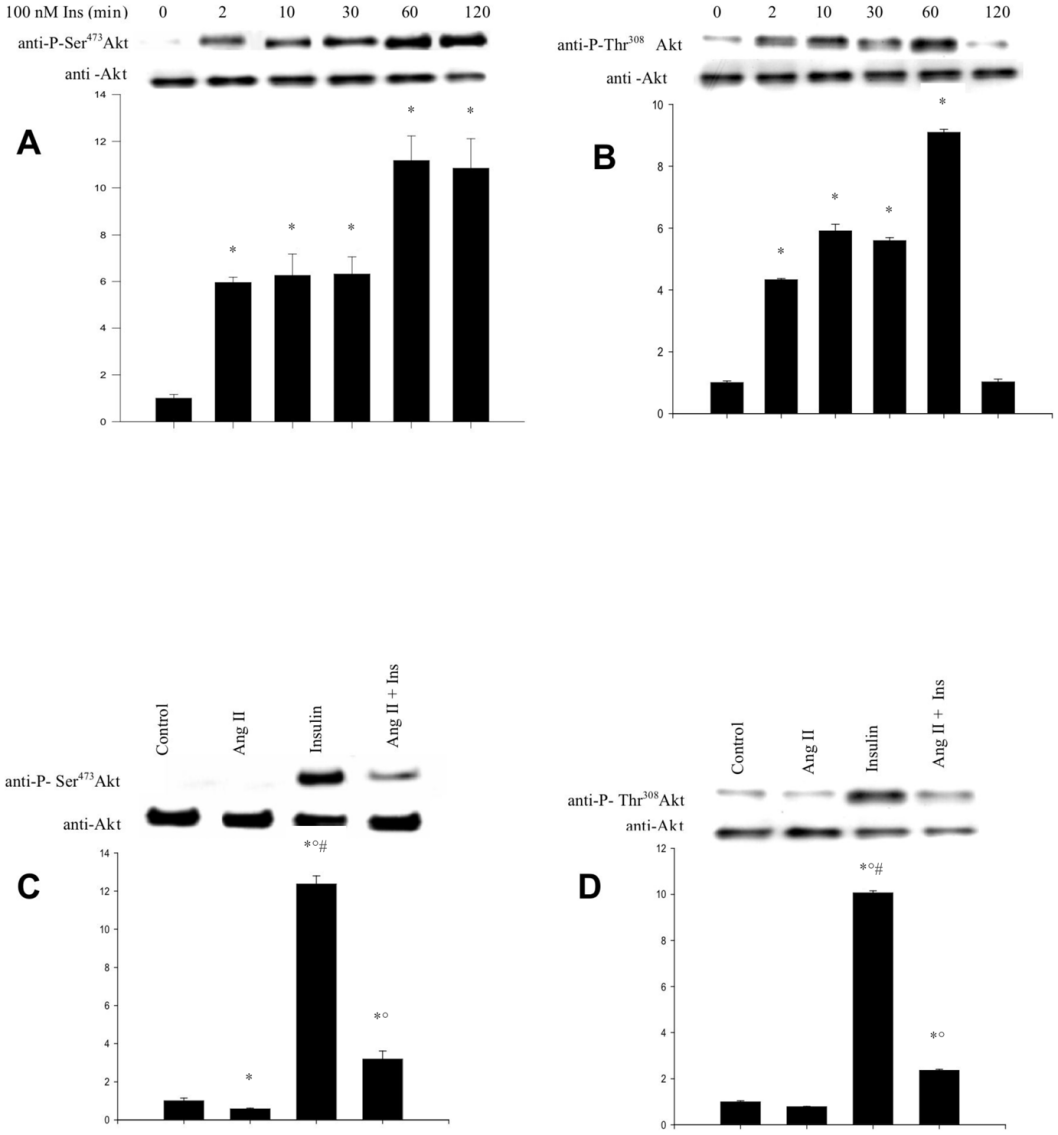
Kinetics of insulin-induced Akt phosphorylation and inhibitory effect of Ang II on insulin-induced Akt activation. L6 myotubes were exposed to insulin (100 nM) for 0–120 min. Blots were probed with anti-Akt and anti-phosphoSer^473^-Akt (***A***) or anti-phosphoThr^308^-Akt (***B***). L6 myotubes were pretreated with Ang II 10 nM for 30 min and stimulated with insulin (100 nM) for 60 min. Blots were probed with anti-Akt and anti-phosphoSer^473^-Akt (***C***) or anti-phosphoThr^308^-Akt (***D***) antibodies. Results are expressed as fold change of the ratio of phospho-Akt/Akt over controls (means ± s.d.) and the blots are representative of three independent experiments (*P<0,05 vs control, °*P*<0,05 vs Ang II, ^#^ *P*<0,05 vs Ang II + insulin).

As expected, treatment of myotubes with 100 nM insulin induced phosphorylation of Akt on both residues in a time-dependent manner with a maximum reached within 60 min of incubation (Fig. 2*A* and *B*). Interestingly, phosphorylation of Ser^473^ was more persistent than that of Thr^308^ (≥120 min. vs. 60 min.), suggesting partial inactivation of the enzyme after 1 hour. Incubation of the myotubes with 10 nM Ang II alone decreased Akt phosphorylation on Ser^473^ below control levels and inhibited insulin-mediated phosphorylation on both Ser^473^ and Thr^308^ by more than 80% (Fig. 2*C, D*). The magnitude of this effect strongly suggests that the inhibition of Akt phosphorylation plays a major role in the impairment of GLUT4 translocation by Ang II.

### Effect of ROS and RNS modulators on the inhibition of insulin-mediated phosphorylation of Akt by Ang II

Since NAD(P)H-oxidase and NOS inhibitors and a free radical scavenger significantly restored Ang II-induced inhibition of GLUT4 translocation to the plasma membrane (Fig. 1*A*), we examined the effect of sensitivity of these ROS and RNS modulators on Ang II-mediated inhibition of Akt phosphorylation in order to verify whether the pathway involved might be the same. As expected, 1400 W, AEBSF and myricetin almost completely suppressed Ang II-induced inhibition of insulin-mediated Akt phosphorylation on Ser^473^ (Fig. 1*B*). Identical results were obtained for Thr^308^ phosphorylation (data not shown). These data indicate that, as observed for GLUT4 translocation, NO and O_2_^−.^ generation are required for the inhibition of Akt phosphorylation by Ang II. Likewise Ang II inhibited basal Akt phosphorylation below control levels (P<0.05) and the ROS and RNS modulators used restored basal phosphorylation (data not shown).

### Nitration of Akt in response to Ang II

NO and O_2_^−.^ are known to react yielding the RNS peroxynitrite. The absolute requirement of NO and O_2_^−.^ generation for the inhibition of Akt phosphorylation (Fig. *1B*) and the subsequent translocation of GLUT4 (Fig. 1*A*), prompted us to verify whether Akt might be a direct target of this reactive species.

**Figure 3:**
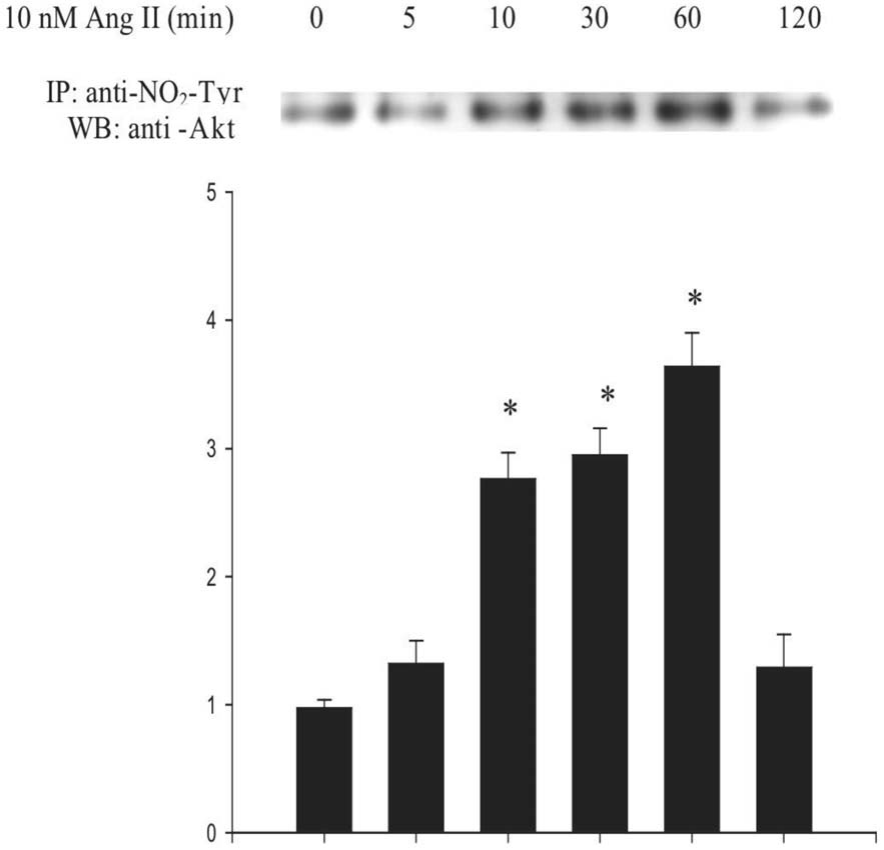
Kinetics of Ang II-mediated Akt nitration. L6 myotubes were exposed to Ang II (10 nM) for 0–120 min. Western blot of nitrated proteins from the soluble fractions were immunoprecipitated with anti-nitrotyrosine antibody and probed with anti-Akt antibody. Results are expressed as the fold increase over controls (means ± s.d.) and the blot is representative of three independent experiments (*P<0,05 vs. control).

As shown in Fig. 3, exposure of L6 myotubes to 10 nM Ang II, resulted in the ability of an anti-nitrotyrosine antibody to immunoprecipitate Akt. This tyrosine nitration was time-dependent, occurring within 10 minutes and peaking at 60 min (approximately 4-fold increase as compared to control), thus indicating a significant nitration of the enzyme in response to Ang II. Nitration levels were back to control values within 120 min. These observations thus confirm the hypothesis that Ang II induces the generation of NO and O_2_^−.^ in myotubes resulting in the formation of peroxynitrite. They also indicate that Akt is a target of this reactive species and is thus a novel nitroprotein.

### Effect of ROS and RNS modulators on insulin-stimulated Akt activity *in cellulo*

Although phosphorylation of Ser^473^ and Thr^308^ residues is generally used as a marker of Akt activation, we directly measured its enzymatic activity by determining its ability to phosphorylate GSK3α which is one of its known targets. L6 myotubes were incubated with the hormones and modulators used previously and Akt was immunoprecipitated. The immunoprecipitated Akt was incubated in vitro with recombinant GSK3α inthepresenceofATPand the phosphorylation of this kinase on Ser^21^ was determined by immunoblotting.

**Figure 4:**
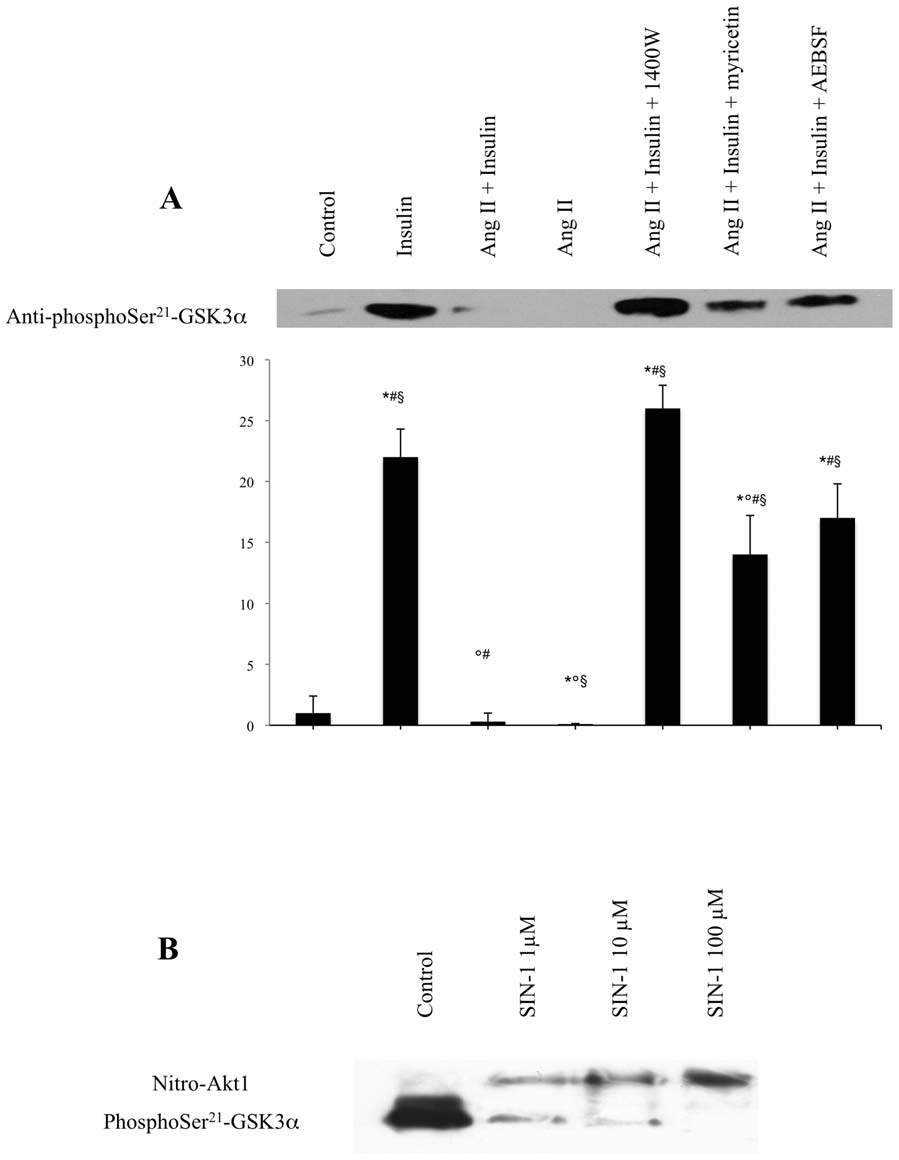
Effect of ROS and RNS modulators and of nitration on Akt ativity. ***A***: L6 myotubes were pretreated with either AEBSF (0.5 mM) for 30 min, 1400 W (10 µM) for 60 min or myricetin (100 µM) for 30 min prior to addition of Ang II (10 nM) for 30 min and subsequent stimulation with insulin 100 nM for 60 min. The activity of Akt immunoprecipitated from the cell lysates was determined by its ability to phosphorylate GSK3α. Western blots were probed with anti-phosphoSer^21^-GSK3α. The blot displayed is representative of three independent experiments. Results are expressed as the fold increase over controls (means ± s.d.) (*P<0,05 vs control, °*P*<0,05 vs Insulin, ^#^ *P*<0,05 vs Ang II, ^§^ *P*<0,05 vs Ang II + Insulin). ***B***: Recombinant human PKB/Akt1 (1.5 µg) was nitrated with SIN-1 (1, 10 and 100 µM) for 1 hour at 30°C, immunoprecipitated and incubated with GST-GSK-3α peptide in the presence of Mg^++^/ATP for 2 hours. Blots were probed with a polyclonal rabbit anti-phosphoSer^21^-GSK-3α and a monoclonal mouse anti-nitrotyrosine antibody. The blots are representative of three independent experiments.

As shown in Fig. 4*A*, insulin (100 nM) stimulated Akt-mediated GSK3α phosphorylation approximately 10-fold and this effect was totally blocked by Ang II. Ang II by itself reduced GSK3α phosphorylation below control levels (P<0.05) as observed for Akt phosphorylation (Fig. 2*C & D*). Likewise, the ROS and RNS modulators alleviated the inbitory effect of Ang II on insulin-mediated activation (Fig. 4*A*). Interestingly, whereas the NADP(H)-oxidase inhibitor AEBSF and the ROS scavenger myrecitin only partly restored Akt activity, the NOS inhibitor 1400 W completely obliterated the effect of Ang II.

These data show that there is an excellent agreement between the phosphorylation of Akt on Ser^473^ and Thr^308^ and its activity as determined by its ability to phosphorylate GSK3α (Fig. 2). They also indicate that the inhibitory effect of Ang II involves a mechanism preventing not only phosphorylation of Akt on Ser^473^ and Thr^308^ but also its catalytic activity.

### In vitro nitration of Akt inhibits its activity

These observations together with those showing that Akt is nitrated in response to Ang II (Fig. 3), raised the question as to whether this nitration directly affects Akt activity or whether it is rather an associated phenomenon which may have other functions, the inhibition being then due to the nitration of other more upstream targets.

In order to test this, we nitrated recombinant Akt in vitro using SIN-1, a compound which spontaneously degrades into NO and O_2_^−.^ yielding peroxynitrite. Nitrated Akt was incubated with recombinant GSK3α whose phosphorylation on Ser^21^ in vitro was determined by immunoblotting.

As shown in Fig. 4*B*, non-nitrated recombinant phospho-Akt heavily phosphorylated GSK3α, whereas prior exposure to SIN-1 inhibited this in a dose-dependent manner. In order to further verify whether the inhibitory effect of the peroxynitrite donor was indeed linked to Akt nitration, the blots were probed with an anti-nitrotyrosine antibody which labelled a band corresponding to Akt only when SIN-1 was added (Fig. 4*B*). The degree of Akt nitration was dose-dependent and as expected, inversely related to GSK3α phosphorylation. This observation clearly indicates that Akt nitration is sufficient to fully inhibit its activity.

### Inhibition of MEK restores insulin-stimulated phosphorylation of Akt in the presence of Ang II

**Figure 5:**
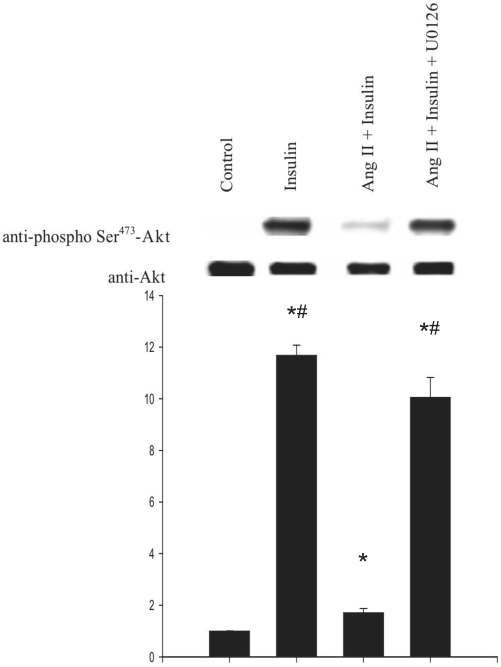
Effect of the MEK inhibitor U0126 on insulin-stimulated Akt phosphorylation. L6 myotubes were treated with U0126 (10 µM) for 30 min prior to addition of Ang II (10 nM) for 30 min with subsequent stimulation by insulin 100 nM for 60 min. Western blots were probed with anti-phosphoSer^473^-Akt and anti-Akt antibodies. The blot is representative of three independent experiments. Results are expressed as the fold increase of the ratio of phospho-Akt/Akt over controls (means ± s.d.). (*P<0,05 vs control, ^#^ *P*<0,05 vs Ang II + Insulin).

The observation that nitration inhibits Akt does however not preclude the possibility that other pathways depending on NO and O_2_^−.^ and therefore probably on ONOO^−^ generation, may be involved in the inhibitory action of Ang II. Among these, ERK 1/2 have been reported to inhibit Akt activation [26]. As we have previously shown Ang II to activate ERK1/2 in an NO and O_2_^−.^-dependent fashion which proved to be the result of their nitration [17]. We therefore verified whether this pathway might also be involved in Ang II-mediated Akt inhibition in myotubes. In order to test this we inhibited the upstream ERK kinase MEK with the specific inhibitor U0126. As shown in Fig. 5, U0126 added to the cells prior to treatment with Ang II and insulin, totally restored insulin-induced Akt phosphorylation, indicating that this cascade is involved in the inhibition of Akt by Ang II in this model. U0126 had no effect on basal or insulin-stimulated Akt phosphorylation (data not shown).

### Ang II induces nitration and NO and O_2_^−.^-dependent activation of ERK 1/2

In order to further verify whether the ERK pathway is indeed involved in the NO and O_2_^−.^-dependent inhibition of Akt, we tested whether their activation by Ang II in these myotubes is accompanied by their tyrosine nitration like in vascular smooth muscle cells [17] and whether it is affected by ROS and RNS modulators.

**Figure 6:**
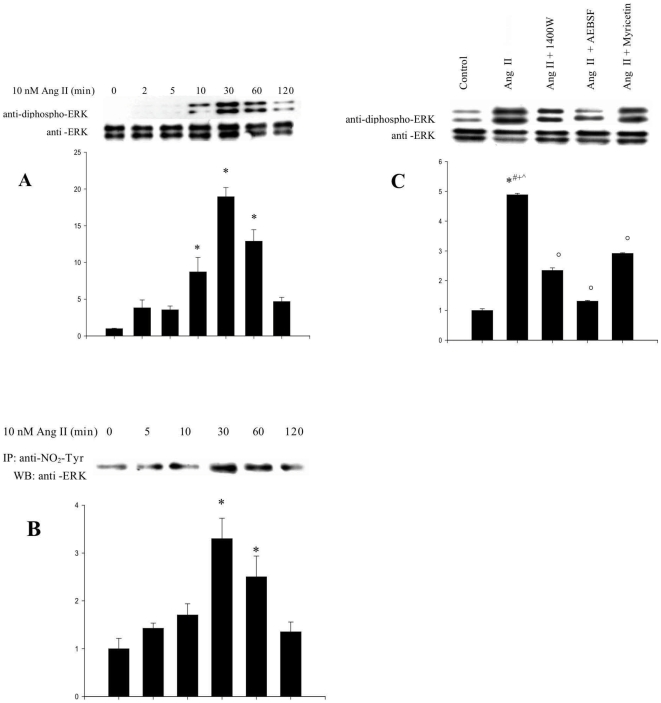
Kinetics of Ang II-dependent phosphorylation and nitration of ERK 1/2. L6 myotubes were exposed to Ang II (10 nM) for 0–120 min. (***A***) Blots were probed with anti-phosphoERK1/2 and anti-ERK1/2 antibodies. (***B***) Samples were immunoprecipitated with anti-nitrotyrosine antibody and probed with anti-Akt antibody. (***C***) Effect of ROS and RNS modulators on ERK 1/2 phosphorylation. L6 myotubes were pretreated with either AEBSF (0.5 mM) for 30 min, 1400 W (10 µM) for 60 min or myricetin (100 µM) for 30 min, prior to addition of Ang II (10 nM) for 30 min and subsequent stimulation with insulin 100 nM for 60 min. Western blots were probed with anti-phosphoERK and anti-ERK antibodies. The blots are representative of three independent experiments. Results are expressed as the fold increase of the ratio of phospho-ERK/ERK over controls (means ± s.d.) (*P<0,05 vs control, °*P*<0,05 vs Ang + Insulin, ^#^ *P*<0,05 vs 1400 W + Ang, *^+^P*<0,05 vs AEBSF + Ang, *^∧^P*<0,05 vs Myricetine + Ang).

Fig. 6*A* shows that Ang II is able to activate ERK 1/2 in L6 myotubes, as determined by its ability to induce their phosphorylation on Thr^202/183^ and Tyr^204/187^ with characteristic kinetics. This activation is accompanied by their nitration following the same kinetic pattern (Fig 6*B*). Further confirming the relationship between ERK nitration and activation, the ROS modulators 1400 W, AEBSF and myrecitin all significantly inhibited Ang II-mediated phosphorylation.

Together with the finding that the MEK inhibitor U0126 alleviates Ang II-mediated Akt inhibition, these observations clearly indicate that the ERK pathway contributes to the inhibition of Akt in this model.

### Mapping of nitrated tyrosines in ERK1

**Figure 7:**
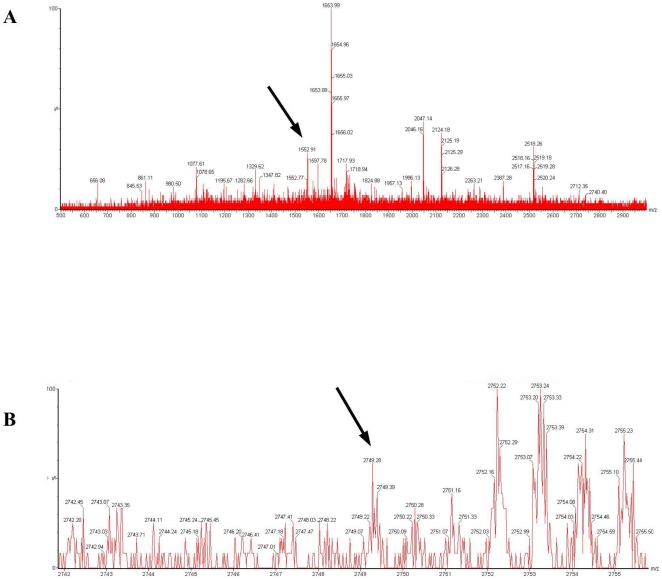
Mass spectra of tryptic peptides of nitrated ERK1. Tryptic peptides of nitrated recombinant ERK1 were immunoprecipitated with anti-nitrotyrosine antibodies. ***A***: Mass spectrum (500–3000 Da) shows a major peak with a monoisotopic mass of 1552.91 corresponding to GLKY^156^-(NO2)IHSANVHR. ***B***: Detailed spectrum (2742–2755.5 Da) shows a major peak with a monoisotopic mass of 2749.28 corresponding to the peptide ASTLEAMRDVYIVQDLMETDLYK containing one nitrated Tyr, Tyr^119^ or Tyr^130^. Other peaks correspond to peptides which do not contain Tyr and were adsorbed non-specifically to the anti-nitrotyrosine agarose beads.

As the molecular mechanism through which tyrosine nitration regulates kinase activity is still unclear, we tried to map the nitration sites in both ERK 1 and Akt1 in order to see whether their location may give some clues. Both recombinant human enzymes were nitrated in vitro with SIN-1 and digested with trypsin and chymotrypsin. The tryptic fragments were immunopurified with an anti-nitrotyrosine antibody and analyzed by MALDI-TOF mass spectrometry. Whereas we were unable to analyze nitrated Akt peptides due to digestion and ionization problems, we identified two peptides displaying a mass difference of +45 by MS analysis, corresponding to the adjunction of a nitro group with ERK1. The peptides with monoisotopic masses of resp. 1552.91 Da and 2749.28 Da correspond to aminoacids 153 – 165 (GLKYIHSANVLHR) and 109–131 (ASTLEAMRDVYIVQDLMETDLYK). The first peptide contains only one Tyr (Fig. 7*A*) indicating a nitration site on Tyr^156^, corresponding to Tyr^139^ in ERK2, whereas the second peptide contains two Tyr residues of which only one was nitrated as indicated by the difference in mass (Fig. 7*B*). We were unable to obtain a complete fragmentation of this peptide for sequencing and could therefore not determine so far which of Tyr^119^ or Tyr^130^ corresponds to the nitration site in this sequence. No corresponding site exists in ERK2. Interestingly, Tyr^156^ which is conserved in ERK2 (Tyr^139^), is located in the α-helix E, close to β6 and the C-loop which contains the active site Asp^166^ with which it might interact.

## Discussion

Since the CAPPP trial reported in 1999 that an ACE inhibitors significantly reduce the risk of developing diabetes as compared to other antihypertensive treatments (β-blockers, diuretics or a combination of both), numerous other trials have confirmed the unique diabetes-preventive properties of ACE inhibitors and Ang II receptor antagonists as compared to other available blood pressure lowering drugs [2], [3], [4], [5], [27]. These trials have prompted research in this area and numerous authors have since reported the ability of these drugs to restore or at least improve insulin sensitivity in diabetic animal models [1], [28] and human subjects [3], [7], [15], [27], [29], [30]. Several reports indicate that the anti-insulinic actions of Ang II are mediated, at least in part, through increased generation of ROS, in particular O_2_^.−^ [1], [8], [11], [15], [31], [32], [33]. Although the pathogenesis of type 2 diabetes is obviously complex and multifactorial, there is evidence that oxidative stress plays an etiologic role as it is associated with hyperglycemia, hypertriglyceridemia, obesity, and hypertension [1], [14], [15], [31], [34] which all occur in this disease. Moreover, NAD(P)Hoxidases which contribute to O_2_^−^ generation are upregulated in type 2 diabetes [34], [35].

Whereas oxidative stress usually evokes the activation of the O_2_^.−^- H_2_O_2_ - OH^.^ generation cascade, O_2_^−.^ can also give rise to the generation of RNS such as N_2_O_3_, NO^+^, NO_2_^.^ and ONOO^−^, depending upon the NO/O_2_^−.^ ratio [21]. Accordingly, the deleterious effects of iNOS, which generates high levels of NO, on insulin sensitivity have been demonstrated more than a decade ago by the seminal work of Marette and coworkers [36], [37]. ONOO^−^ which can affect DNA, lipids and proteins [20], [21], gives rise to nitrative stress which has gained much interest during the past few years as it has been reported to be involved in various signaling pathways and hence in a growing number of diseases [20], [38], [39], [40] including diabetes [18], [19], [25], [41], [42]. Peroxynitrite has been shown to nitrate tyrosine residues and consequently modify the functional properties of several proteins [17], [20], [21], [38], [43]. Increased protein nitration has been shown to occur in the mitochondria of diabetic mice [19] and nitrated proteins have been found in plasma of diabetic patients [41], [42], [44].

Interestingly, Ang II has been shown to stimulate the generation of peroxynitrite [33], [39], [45], [46], [47] and ARBs have been reported to selectively inhibit iNOS and NAD(P)Hoxidase expression and activity as well as nitrative stress in both type 1 and 2 diabetic animal models [1], [14], [32], [33]. Confirming the significance of ONOO^−^ generation, we have shown that Ang II stimulates the nitration of MEK 1 and ERK 1/2 [17].

These observations have prompted us to investigate the potential role of Ang II-mediated nitration on the metabolic pathways of insulin involved in glucose uptake in skeletal muscle. We report here that Ang II impairs insulin-mediated GLUT4 translocation (Fig. 1*A*) and inhibits the phosphorylation of Akt (Fig. 2*C & D*) which acts as a common upstream activator of most of insulin's metabolic actions including glucose uptake. Previous reports have shown that Ang II is able to modulate insulin-stimulated Akt activity and have suggested that this involves increased production of ROS [1], [6], [48]. We show here that in L6 myotubes, a skeletal muscle model, Ang II induces the nitration of Akt (Fig. 3) and that this nitration inhibits the kinase's activity (Fig. 4). In agreement with a regulation of Akt by RNS, previous reports indicate that Akt is inhibited by S-nitrosation of its Cys^22^ in insulin resistance [23], [24]. S-nitrosylation or nitrosation is the modification of sulfhydryl (-SH) to S-nitroso (-SNO) groups and appears to occur at a molar NO/O_2_^.−^ ratio of 2 to 3 [49], which probably corresponds to the physiological redox status [21], [50]. S-nitroso groups are unstable and protein S-nitrosation is therefore a rapidly and spontaneously reversible posttranslational modification. Interestingly, one of the reports indicates that increasing the level of O_2_^.−^, also increased the inhibition of Akt by the pure nitric oxide donor SNAP [23]. Although the authors did not investigate the potential tyrosine nitration of Akt under these conditions, it was most likely to occur since increasing concentrations of O_2_^.−^ in the presence of NO tends to shift the production of nitrosating species (NO^+^ and/or N_2_O_3_) towards peroxynitrite which selectively nitrates tyrosine residues [20], [21], [49], [50]. One can thus reasonably speculate that these latter conditions generated a nitrative rather than or parallel to a nitrosative stress. Since Ang II has been shown to induce oxidative and nitrative stress at physiological and low nanomolar concentrations, tyrosine nitration rather than S-nitrosation appears to be the most likely mechanism of Akt inhibition by this peptide in our model. This hypothesis is confirmed by our data which show firstly the restoration of its activity by the blockade of NO and O_2_^.−^ generation (Fig. 4*A*) and secondly, its tyrosine nitration and consequent loss of catalytic activity (Fig. 3 & 4*B*). In addition, whereas the effect of compounds acting either on the production or the scavenging of O_2_^.−^, like the NAD(P)Hoxidase inhibitor AEBSF and the scavenger myricetin, indicate that increased oxidative stress is involved in this process (Fig. 4*A*), the effect of the selective iNOS inhibitor 1400 W (Fig. 4*A*), shows that nitric oxide is also required, confirming that Akt inhibition depends on the reaction products of both O_2_^−.^ and nitric oxide and not solely on O_2_^.−^.

As indicated above, S-nitrosation is a modification which is rapidly reversible by reduction and which therefore probably is a fine-tuning mechanism of enzyme activity depending upon small variations of O_2_^.−^ production which is well below that of NO under physiological conditions [21], [51]. Conversely, peroxynitrite generation requires equimolar concentrations of O_2_^.−^ and therefore only occurs when specific generating enzymes or systems, e.g. NADP(H)-oxidases, xanthine oxidase, mitochondrial complexes I and III or iNOS, are activated. This occurs in response to various signals including hormones and cytokines as well as pathological conditions like hypoxia, ischemia, hypo- and probably hyperglycemia [20], [34], [38], [40], [42]. In addition, tyrosine nitration is stable and most likely requires an enzyme to be reversed [52], [53]. It is therefore much more likely to exert deleterious effects than S-nitrosation. As an example of this hypothesis, we have recently reported that albumin is nitrated in newborns who suffered perpartal asphyxia and that the levels of plasma nitroalbumin are highly correlated to the severity of the ensuing cerebral lesions [40].

Although the intramolecular interactions involved in the phosphorylation and activation of Akt are extremely complex, the interactions of its pleckstrin homology (PH) domain and the hydrophobic motif (HM; Phe^469^-X-X-Phe^472^-Ser^473^-Tyr^474^) of its C-terminal part with the kinase domain appear to play a crucial role [54]. It has been proposed that the HM plays a dual role in the regulation of Akt activity depending upon either its binding to Trp^80^ or its burying in the hydrophobic groove. Interestingly, this motif contains a Tyr residue at position 474. It is therefore conceivable that if this Tyr were to be nitrated, it may switch the kinase from the active to the inactive state by increasing its hydrophilicity. Moreover, this Tyr has also been reported to be phosphorylated, this modification being required for the full activation of Akt [55]. There are however 15 other Tyr residues in Akt1, 3 of which are located in the PH domain and 11 in the kinase domain. Among the latter is Tyr^272^ located next to Arg^273^ which is believed to be in contact with the primary phosphate of ATP and to Asp^274^ which is assumed to be the active site [56]. Nitration of this Tyr might thus also interfere with ATP binding. Obviously, mapping of the nitrated Tyr residue(s) will be necessary to understand the intramolecular interactions resulting from this posttranslational modification.

As stated earlier, direct nitration of Akt does however not preclude the existence of other pathways contributing to its inactivation by Ang II.

Among the other pathways which might potentially be involved in the inhibition of Akt activity, Sinha et al. previously reported the involvement of the ERK through the formation of an inhibitory complex with Rsk and PDK1-bound Akt in renal tubular cells [26]. Another group reported that Ang II inhibits insulin-mediated glucose uptake in VSMC through Ser^307^ and Ser^616^ phosphorylation of insulin-receptor substrate-1 by ERK and JNK [57]. Since we had previously shown that Ang II-mediated ERK activation in VSMC requires its nitration [17], we tested their putative involvement in skeletal muscle cells. We found the MEK inhibitor U0126 to almost completely restore insulin-dependant Akt activation in the presence of Ang II (Fig. 5), thus indicating the contribution of the MEK – ERK pathway to the inhibition of Akt in this model. Interestingly, enhanced ERK activation has also been reported in skeletal muscle of women with polycystic ovary syndrome where it has been found to be linked to insulin resistance [58]. A third potential pathway involving ERK has been proposed by Skidgel's group. They reported that in endothelial cells, kinin B1 receptors activate iNOS via ERK-mediated phosphorylation of its Ser^745^ [59], [60]. This pathway may obviously result in increased ONOO^−^ generation and hence, potentially in Akt nitration. This pathway is likely to be involved in our model as well, since inhibition of MEK almost completely restores Akt activity (Fig. 5). Further supporting this hypothesis, is the fact that we found nitration to be sufficient to completely inhibit recombinant phosphoSer^473^ Akt which is partly active [61] (Fig. 6B).

**Figure 8:**
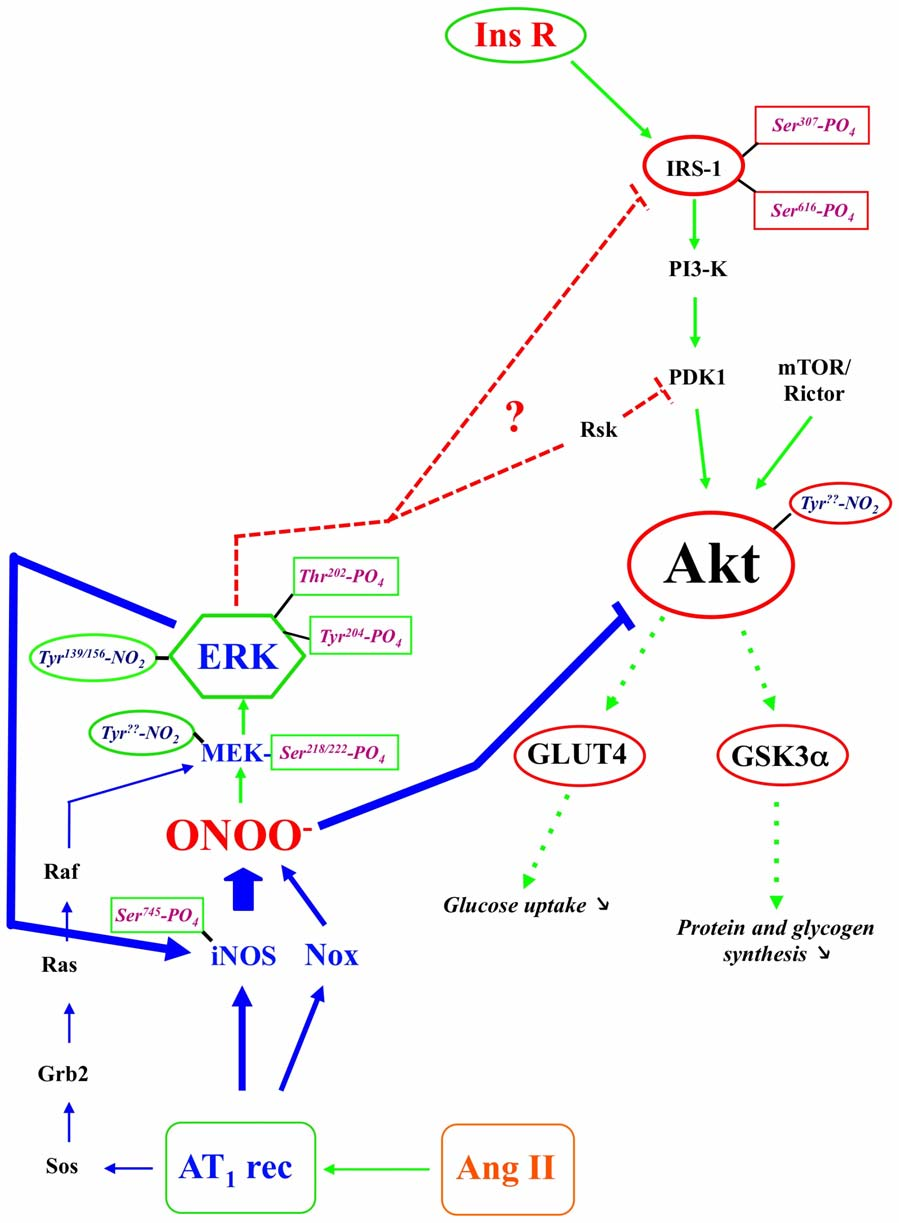
Schematic representation of the putative mechanisms involved in the regulation of Akt activity by insulin and Ang II. Activating pathways are shown in green and inhibitory mechanisms in red.

Similar to VSMC [17], we found Ang II-mediated ERK activation to require tyrosine nitration as it is abolished by 1400 W, AEBSF, and myricetin in myotubes (Fig. 6*C*). We mapped the nitration sites of ERK1 and found them to correspond to Tyr^156/139^, located in the kinase domain of both ERK1 and ERK2, and Tyr^119^ or Tyr^130^, expressed only in ERK1 (Fig. 7). These observations thus further support the hypothesis that nitration plays a major role in Ang II-mediated Akt inhibition (Fig. 8) even if they do not rule out the potential role of other oxidative processes such as S-nitrosation.

These observations all point towards a major role of nitrative stress and protein nitration in the pathogenesis of insulin resistance and its complications. Our data provide two novel targets, ERK1/2 and Akt, of a pathological nitrative stress resulting in the impairment of glucose uptake in skeletal muscle. In agreement with cell and animal studies as well as clinical trials they also confirm the deleterious effects of Ang II which acts both in a chronic fashion by increasing the expression of NAD(P)Hoxidase and iNOS and in acute manner by activating them thereby leading to increased nitrative stress. The activation mechanisms of iNOS are obviously very complex and involves interactions with multiple proteins [62] including Rac2 [63], Src [64] and Gα subunits [65] which can be triggered by Ang II AT_1_ receptors. The recent reports by Skidgel's group [59], [60] suggesting the involvement of ERK are supported by our observations and further strengthen the role of this enzyme in G-protein coupled receptor signaling pathways such as Ang II and kinins. Interestingly, ACE inhibitors, which inhibit Ang II production from Ang I, also increase the levels of kinins, which are normally degraded by ACE. Skidgel's findings therefore imply that ACE inhibitors, which are also allosteric agonists of B_2_R receptors, may result in the inhibition of Akt through the activation of iNOS. Whether this effect outweighs the inhibition of the Ang II-dependent effects remains to be determined, but if this is indeed the case it would suggest that AT_1_ receptor antagonists would be superior to ACE inhibitors in preventing or reducing insulin resistance. So far, clinical trials comparing both classes of drugs have failed to show significant differences on glucose metabolism [66], [67], [68], but more sophisticated analysis of glucose tolerance in these patients would be required to verify this.

Whereas ARBs and ACEI have demonstrated their efficiency in improving insulin sensitivity and reducing end-organ damage and are now often considered as first-line therapy in hypertension with metabolic syndrome or diabetes [3], [4], [5], [30], association of anti-oxidants or anti-nitrants in an attempt to further reduce deleterious nitrative stress might be worth considering in severely affected or non-responsive patients.

## Materials and Methods

### Reagents

Ang II was from Bachem, myricetin, SIN-1 and nitro-l-arginine-methyl-ester (l-NAME) from Cayman, insulin and 3-nitrotyrosine were purchased from Sigma. 4-(2-Aminoethyl) benzenesulfonyl fluoride (AEBSF) and Chemiluminescence reagent were from Uptima, Interchim. 1400 W and Trolox were from Acros Organics. The MEK inhibitor U0126 was from Cell Signaling Technology. Active recombinant human PKB/Akt1 was from Assay Designs and the Akt activity immunoassay kit from Calbiochem. Dulbecco's modified Eagle's Medium (containing 1 g/L glucose, pyruvate and glutamax I), fetal bovine serum (FBS), horse serum (HS) and other cell culture reagents were purchased from Invitrogen. Cell culture dishes were from Nunc. All other reagents were of analytical grade.

### Antibodies

Antibodies directed against Akt, phosphoSer^473^-Akt, phosphoThr^308^-Akt and diphosphoERK (ERK1/2) were purchased from Cell Signaling Technology. Anti-ERK1/2 antibody was from Sigma. Anti-GLUT4 antibody was from Biogenesis. Affinity-purified polyclonal anti-nitrotyrosine antibodies were obtained as described previously [40]. A monoclonal anti-nitrotyrosine antibody (clone 2E11) was obtained from Antibodies-online. Anti-HRP-conjugated anti-rabbit and anti-mouse IgG antibodies were from Jackson Immunology Laboratories®.

### Cell Culture

L6 rat skeletal muscle cells (American Type Culture Collection) were grown in DMEM (1 g/L glucose) containing 20% (v/v) of fetal bovine serum (FBS) and gentamycin (10 µg/ml) at 5% CO_2_ in 37°C until 80–90% confluence. Myoblasts were differentiated into myotubes by replacing FBS with 2% horse serum for 6–8 days. Cells of passages 3–6 were used for experiments.

### GLUT4 translocation

Subcellular fractionation of myotubes was carried out as described by Yonemitsu et al. [69] with slight modifications. The cells from 10-cm dishes were gently scraped, centrifuged (1000 x *g* for 10 min) at 4°C. All subsequent steps were carried out at 4°C. Cells were resuspended in buffer containing 250 mM sucrose, 5 mM NaN3, 2 mM EGTA, 20 mM HEPES pH 7.4, and a protease inhibitor cocktail, as described above, and homogenized using 20 strokes of a Dounce homogenizer. The homogenate was centrifuged at 760 x *g* for 5 min to remove nuclei and unbroken cells. The supernatant was centrifuged at 30000 x *g* for 60 min to pellet the crude plasma membrane fraction. Plasma membrane and total cell lysate proteins were resolved by SDS-PAGE, blotted on PVDF membrane and probed with anti-GLUT4 antibody.

### Immunoprecipitations and immunoblotting

For immunoprecipitation, 50 µg of cell lysate protein was incubated overnight at 4°C with anti-nitrotyrosine antibody covalently coupled to agarose beads according to the manufaturer's protocol (CarboLink, Pierce**®**). Covalent hydrazone bonds were stabilized by reduction with NaCNBH_3_. For immunoblotting, samples were subjected to 10% SDS-PAGE and transferred to a PVDF membrane (Perkin Elmer Life Sciences Inc**®**.). Membranes were blocked with TBST (Tris 50 mM, NaCl 100 mM, pH 7.4, Tween 20 0.1%) containing 5% milk and probed with the antibodies indicated in the figure legends and appropriate HRP-labeled secondary antibodies. The blots were revealed by enhanced chemiluminescence. Densitometric analysis was performed using Scion Image software.

### PKB/Akt activity assays

PKB/Akt activity assays were performed according to the manufacturer's protocol using either 100 µg of L6 cell lysate protein or 0.5 µg of recombinant human active PKB/Akt1 nitrated with SIN-1 in buffer containing 50 mM Tris (pH 7.4) and 100 mM NaCl for 1 hour at 25°C. Active recombinant Akt1 corresponds to Akt1 purified from extracts of Sf9 cells co-expressing full-length human Akt1 and mTOR.

Briefly, PKB/Akt was immunoprecipitated and incubated with a GST fusion protein comprising the GSK-3α phosphorylation sequence in the presence of Mg^++^/ATP. Samples were subjected to 12% SDS-PAGE and transferred to a PVDF membrane (Perkin Elmer Life Sciences). Membranes were blocked with TBST (Tris 50 mM, NaCl 100 mM, pH 7.4, Tween 20 0.1%) containing 5% milk and probed with an anti-phosphoSer^21^-GSK-3α followed by an HRP-labeled anti-rabbit antibody. The blots were revealed by enhanced chemiluminescence.

### Mapping of nitrated tyrosine residues by mass spectrometry analysis

Recombinant human ERK1-GST (Upstate Biotech) was nitrated with 100 µM SIN-1 in buffer containing 20 mM MOPS (pH 7.2), 25 mM β–glycerol phosphate, 5 mM EGTA, 1 mM sodium orthovanadate, with 15 mM MgCl_2_ and100 µM ATP for 2 hours at 20°C. After nitration, the GST was cleaved with thrombin-agarose (Sigma**®**) according to the manufacturer's protocol. GST was removed by glutathione-agarose (Pierce**®**) affinity chromatography. The nitrated ERK samples were digested with trypsin (Promega**®**) at 25 ng/ml during 2 h. The samples were diluted in 25 mM ammonium bicarbonate (pH 7.4) with 50 mM NaCl and then incubated in the presence of 20 µl agarose-conjugated anti-nitrotyrosine antibodies. The nitrated peptides were eluted with 50% acetonitrile/0.2% trifluoroacetic acid, dried, resuspended in 1.5 µl of matrix suspension (4 mg α-cyano-4-hydroxycinnamic acid, 0.8 mg 3,5-dihydroxybenzoic acid in 50% acetonitrile/0.1% trifluoroacetic acid, 0.1 mM fucose), and deposited on a stainless steel target. Mass spectrometry analysis was performed on a Q-TOF Ultima Global mass spectrometer (Micromass**®**) equipped with a MALDI source. Ionization was achieved using a nitrogen laser (337 nm beam, 10 Hz). The instrument was externally calibrated using the monoisotopic masses of tryptic and chymotryptic peptides from bovine serum albumin. Acquisitions were performed in a V-mode reflectron position. Microsequencing was performed by argon-induced fragmentation after selection of the parent ion.

### Statistical analysis

Statistical analysis was performed by one-way ANOVA followed by the Holm-Sidak method for comparison between groups. P<0.05 was considered to be statistically significant. Error is represented as standard deviation.
